# Effects of Whole‐Body Vibration Therapy on Distal Tibial Myotendinous Density and Volume: A Randomized Controlled Trial in Postmenopausal Women

**DOI:** 10.1002/jbm4.10120

**Published:** 2018-12-13

**Authors:** Miranda K Boggild, George Tomlinson, Marta C Erlandson, Eva Szabo, Lora M Giangregorio, B Catharine Craven, Lubomira Slatkovska, Shabbir MH Alibhai, Angela M Cheung

**Affiliations:** ^1^ Department of Medicine University of Toronto Toronto Ontario Canada; ^2^ Department of Medicine University Health Network Toronto Ontario Canada; ^3^ College of Kinesiology University of Saskatchewan Saskatoon Saskatchewan Canada; ^4^ Department of Kinesiology University of Waterloo Waterloo Ontario Canada; ^5^ Schlegel and University of Waterloo Research Institute for Aging Waterloo Ontario Canada

**Keywords:** AGING, SKELETAL MUSCLE, SARCOPENIA, EXERCISE, THERAPEUTICS, CLINICAL TRIALS

## Abstract

Whole‐body vibration (WBV) therapy has been proposed as a therapy to reduce sarcopenia and improve muscle strength. The purpose of this study was to explore whether 12 months of WBV therapy increases myotendinous density and volume of the distal tibia as measured by HR‐pQCT in postmenopausal women in a parallel group, randomized controlled trial with 1:1:1 allocation to three arms. Postmenopausal women (*N* = 202) with low hip BMD were randomized to 20 min daily of 0.3*g* 30‐Hz WBV therapy, 0.3*g* 90‐Hz WBV therapy using the Juvent platform (Juvent, Somerset, NJ, USA), or no WBV. The main outcome measure was myotendinous density (HU) and volume (mm^3^) at the distal tibia measured at baseline and 12 months with HR‐pQCT. There were no significant effects on myotendinous density or volume at the distal tibia after 12 months of daily 30‐ or 90‐Hz WBV therapy compared with no WBV therapy. Mean change (SD) in myotendinous density from baseline was 4.6 (5.7) HU in the 30‐Hz WBV group, 3.9 (6.1) HU in the 90‐Hz WBV group, and 3.9 (5.4) HU in the control group (*p* = 0.70). Mean change (SD) in myotendinous volume from baseline was −7 (503) mm^3^ in the 30‐Hz WBV group, 111 (615) mm^3^ in the 90‐Hz WBV group, and 35 (615) mm^3^ in the control group (*p* = 0.50). In conclusion, WBV therapy at 30‐ or 90‐Hz for 12 months had no significant effects on myotendinous density or volume at the distal tibia as measured by HR‐pQCT in postmenopausal women. © 2018 The Authors. *JBMR Plus* Published by Wiley Periodicals, Inc. on behalf of the American Society for Bone and Mineral Research.

## Introduction

Sarcopenia, defined as an age‐related loss of skeletal muscle, is a result of multifactorial changes that lead to declines in muscle mass, strength, and function.^1^, ^2^ Sarcopenia is common and is frequently associated with frailty, functional impairments, and an increased propensity for falls and subsequent fractures.^2^, ^3^ There has been great interest in how age‐related muscle loss can be minimized to prevent functional disability, falls, and fractures, as these outcomes can have a significant impact on an individual's morbidity and health‐related quality of life.^4^ One proposed intervention for maintaining lean tissue mass (muscle and tendons) during aging is whole‐body vibration (WBV) therapy.^5^

WBV therapy consists of standing on an oscillating platform that oscillates vertically or sideways at a preset frequency (measured in Hertz) and magnitude (measured in units of acceleration based on gravity, *g* = 9.81 m/s^2^).^6^ Vibrations are transmitted from the feet up through the legs and are hypothesized to have interrelated effects on bone, muscle, and tendon.^(7)^ The therapeutic benefits for specific tissues and potential adverse effects of WBV are linked to the choice of plate, vibration frequency, and magnitude.^7^, ^8^, ^9^

Proposed therapeutic mechanisms of action of WBV include mimicking the impact of exercise by invoking the tonic vibration reflex or stretch reflex while voluntarily standing and simulating regional increases in circulation to bone, muscle, and tendon.^7^ Muscle volume and myotendinous density provide an indication of muscle and myotendinous tissue quality; lower density muscle is associated with higher fat content and lower muscle strength in older adults.^10^

Randomized controlled trials and meta‐analyses investigating the effect of WBV on muscle in older adults have used several measures of muscle and tendon function as outcomes, such as strength, mobility, postural control, balance, and falls.^11^, ^12^, ^13^, ^14^ Currently, there is significant heterogeneity in the existing literature regarding the WBV therapy protocols implemented, the duration of the WBV interventions, and how muscle (strength, volume, cross‐sectional area, and density) and myotendinous function are measured, as well as the findings.^11^, ^12^, ^13^, ^14^ Two studies found a significant increase in muscle mass^15^ or cross‐sectional muscle area^16^ in the upper leg with WBV therapy compared with controls.^15^, ^16^ One study reported a significant increase in muscle density of the vastus medialis in the WBV group.^16^ Using whole‐body DXA scans, Verschueren and colleagues observed a decrease in total fat mass with WBV as well as with resistance training compared with controls in postmenopausal women, but no change in lean body mass.^17^ Other studies by Figueroa et al. have shown an increase in leg strength without an increase in leg lean mass with WBV therapy in postmenopausal women with prehypertension or hypertension.^18^, ^19^ Another study from this group confirmed the increase in leg strength with WBV therapy and found an increase in leg lean mass only when WBV therapy was combined with L‐citrulline supplementation.^20^ In obese postmenopausal women, a decrease in body fat percentage has been seen with WBV therapy.^20^, ^21^ Overall, existing research has not conclusively demonstrated how and if WBV therapy exerts therapeutic effects on bone, muscle, and tendon in older adults.

With aging, not only do we lose bone and muscle mass, but lipid deposits in muscle and myotendinous tissues increase; as a result, muscle and myotendinous tissue density decreases.^10^, ^22^ Understanding these complex interrelationships between fat, muscle, and bone and mechanical stimuli, which share a common progenitor (mesenchymal stem cells), may help us understand the relationships between obesity, sarcopenia, and osteoporosis, which are common comorbidities that contribute to multimorbidity in older adults.

This study aims to determine if 1 year of WBV therapy in postmenopausal women has a beneficial effect on muscle and tendon of the distal tibia region, namely increases in the myotendinous density (M_T_D) and myotendinous volume (M_T_V). This site is clinically relevant for falls.

## Participants and Methods

Our group has previously reported on the bone outcomes (primary outcome) of the Vibration Study (The Influence of Vibration on Bone Mineral Density in Women Who Have Weak Bones After Menopause; NCT00420940), a randomized controlled trial of daily WBV therapy in postmenopausal women, and showed no change in BMD or bone structure after 12 months of therapy.^23^ Here we examine the secondary outcomes, M_T_D and M_T_V, from this trial. The myotendinous characteristics, M_T_D and M_T_V, were analyzed using HR‐pQCT at baseline and after 12 months of daily low‐magnitude (0.3*g*) WBV at either 30‐ or 90‐Hz versus no WBV. The WBV platforms were supplied by Juvent (Somerset, NJ, USA). Calcium and vitamin D supplements were supplied by Jamieson Laboratories (Toronto, Canada). This study was approved by the research ethics board of the University Health Network, Toronto, Canada; all study visits were conducted at this institution as well. Funding for the study was provided by a peer‐reviewed grant from the Ontario Physicians’ Services Incorporated Foundation. These aforementioned industry partners did not participate in the study design, analysis, interpretation, or decision to submit the study for publication.

### Participants

Postmenopausal women were recruited from October 2006 to November 2008 via flyers, word of mouth, and a postmenopausal health newsletter. Informed consent was obtained from all participants. Inclusion criteria were menopause ≥1 year ago and baseline BMD with lowest *T*‐score between −1.0 and −2.5 at the lumbar spine, femoral neck, or total hip. Exclusion criteria were fragility fracture after age 40, diseases or medications that affect bone metabolism or cause bone loss, contraindications to WBV therapy (eg, knee or hip joint replacements), body mass ≥90 kg, expected changes in physical activity levels, or expected travel for >4 consecutive weeks during the study. Further details on BMD inclusion criteria are in the primary study article.^23^

### Randomization and intervention groups

Computer‐generated block‐randomization with a 1:1:1 allocation ratio and a block size of 12 were used to randomize participants to intervention groups. A researcher not involved in the study prepared sealed, sequentially numbered randomization envelopes. The intervention groups were: (1) 0.3*g* 90‐Hz WBV, (2) 0.3*g* 30‐Hz WBV, or (3) control (no WBV). All participants received calcium and vitamin D supplements regardless of intervention group allocation. Researchers conducting myotendinous assessments were blinded to group allocation, but participants were not blinded to the receipt of WBV therapy or control, as we did not use sham WBV. Participants randomized to the WBV groups were given a synchronous 0.3*g* vertical vibration platform that oscillates at 30‐ or 90‐Hz according to their randomized group and asked to voluntarily stand with hips and knees extended on the platform for 20 min daily for 12 months. Instructions for posture while on the platform included standing erect with neutral posture in the neck, spine, and knees; without wearing shoes; and without excessive movement. Participants in the control group were asked not to use WBV therapy. All participants at baseline, and at 6 and 12 months completed a recall questionnaire regarding their calcium and vitamin D intake^24^ and were given supplements to reach a total daily intake from all sources of 1200 mg of elemental calcium and 1000 IU of vitamin D.

The frequency and magnitude of WBV therapy were chosen based on existing literature. Many of the randomized controlled trials looking at muscle outcomes used vertical vibration with a frequency of 20 to 40 Hz.^13^, ^14^, ^16^ We also included a 90‐Hz group to see if higher frequency vibrations would have a greater effect on bone, as postulated from animal experiments.^25^ Low‐magnitude (0.3*g*) WBV therapy was chosen as high‐magnitude vibrations (>1*g*) have a greater theoretical risk of tissue injury or adverse effects.^26^

At 12 months, adherence to the WBV therapy was estimated from the WBV platform's internal digital clock that recorded the date, time, and duration of each of the participants’ WBV sessions. Although the study protocol called for 20 consecutive min daily of WBV therapy, actual use could include single or multiple uses daily <20 min, but none >20 min because the platforms automatically turned off at 20 min. Three different summaries of adherence were calculated: (1) cumulative duration, (2) number of days, and (3) number of 20‐min sessions over the 12‐month period. An adherence self‐report to calcium and vitamin D supplementation was obtained at 12 months; an interim adherence self‐report to WBV therapy and feedback was obtained at 6 months.

### Outcomes and follow‐up

**Figure 1:**
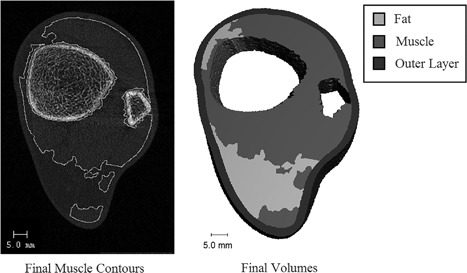
Representative HR‐pQCT scan at the distal tibia. Reprinted from Journal of Clinical Densitometry, vol. 20, MC Erlandson, AKO Wong, E Szabo, N Vilayphiou, MA Zulliger, JD Adachi, AM Cheung, Muscle and myotendinous tissue properties at the distal tibia as assessed by high‐resolution peripheral quantitative computed tomography, pages 226–232, Copyright (2016), with permission from Elsevier.

The distal lower extremity (tibia region) was scanned at baseline and 12 months at the University of Toronto Centre of Excellence in Skeletal Health Assessment, using HR‐pQCT according to the manufacturer's protocol (XtremeCT, Scanco Medical, Zurich, Switzerland). Measurements were done at the left lower leg unless there was a history of fracture or foot drop, in which case the right leg was used. The cross‐sectional anatomy of the distal lower extremity contains bone, muscle, tendon, and fat. The myotendinous tissue was contoured and separated from fat, but as the technique cannot differentiate between muscle and tendon, it is measured together as “myotendinous tissue.” The density (M_T_D) and volume (M_T_V) of this myotendinous tissue were calculated by an algorithm developed and described in detail by our research group in collaboration with Scanco Medical (Soft Tissue Analysis version 1.1).^27^ M_T_V was measured in cubic millimeters (mm^3^) and M_T_D in Hounsfield units (HU). Briefly, the first step of the algorithm was to downscale the images from 82 μm to 164 μm to reduce processing time. Next, the bones and outer skin layer were automatically identified and removed from the region of interest. Myotendinous and fat seed volumes were then identified using tight threshold limits of 100 to 600 HU and −600 to −200 HU, respectively, with volumes of <80 voxels removed to reduce noise. The seed volumes were then iteratively expanded over 10 steps. In each step, the volumes were diluted by one voxel, and volumes where myotendinous and fat boundaries overlapped were set as undefined. Finally, small speckles were removed from the myotendinous and fat regions slice by slice. A representative HR‐pQCT scan at the distal tibia is shown in Fig. 1.^27^

At the baseline and 12‐month study visits, additional information was collected from all participants, including their self‐reported falls, fractures confirmed by radiology reports or radiography, adverse events, and physical activity level based on the Minnesota Leisure‐Time Physical Activity Questionnaire daily activity metabolic index.^28^ Body mass and BMI were measured and the Timed‐Up and Go (TUG) test^29^ administered at baseline and 12 months. If available, serum 25‐hydroxyvitamin D levels from within 3 months of study participation were obtained from medical records.

### Statistical analysis

Our primary analysis was an intention‐to‐treat (ITT) comparison of the 12‐month change from baseline in M_T_D and M_T_V between the intervention groups (90‐Hz WBV versus 30‐Hz WBV versus control groups) using linear regression and adjusting for baseline measurements (ANCOVA). We also assessed three secondary outcomes: TUG^29^ using ANCOVA, and numbers of participants with fractures and falls using Fisher's exact test. The effect of WBV therapy on M_T_D, M_T_V, and TUG were also examined using per‐protocol analyses (defined by completeness of data and adherence). For both myotendinous outcomes and TUG, we specified that the overall *F*‐test of the effect of treatment would be followed by all three two‐way comparisons (90‐Hz WBV versus control, 30‐Hz WBV versus control, and 90‐Hz WBV versus 30‐Hz WBV), with adjustment for multiple comparisons by the single‐step method.^30^ As concurrent physical activity and vitamin D intake could affect myotendinous and fall outcomes and could be different across intervention groups as there was no blinding of group allocation, we compared these variables between groups to assess change in behavior because of treatment. A priori‐selected baseline variables (body mass, height, age, physical activity level, serum vitamin D level, and baseline myotendinous measurements) were assessed for interactions with treatment; these baseline characteristics were used as continuous variables and as dichotomous variables with prespecified cut points. Vitamin D was included because vitamin D deficiency is associated with muscle weakness and sarcopenia,^31^ and meta‐analysis suggests vitamin D supplementation with 1000 IU may prevent falls in older adults.^32^ Where more than 5% of subjects were missing an outcome, we ran the ANCOVA model on five multiply‐imputed data sets^33^; with fewer than 5% missing outcomes, we used single imputation from a regression using the baseline value and randomized group. All analyses were performed using R 3.4.0 (R Foundation for Statistical Computing, Vienna, Austria); a *p* value <0.05 indicated statistical significance.

As myotendinous outcomes had not been previously assessed using HR‐pQCT, we were unable to calculate the power of this study to detect a specified absolute change in M_T_D and M_T_V. The original study was designed to have a high power to detect a difference in bone outcomes with a sample size of 200.^23^

## Results

### Participant characteristics

**Figure 2:**
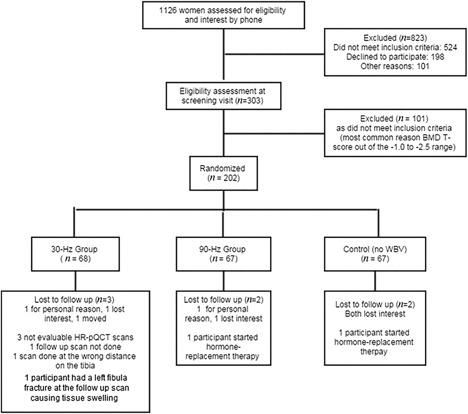
Study flow diagram.

The numbers of participants and study conduct are provided in Fig. 2. All randomized participants were included in the ITT analysis. The mean age of participants was 60 years (range 44 to 79 years; Table 1). The predominant ethnicities of the group were European (78%) and Southeast Asian (16%). Mean body mass was 63 kg with a range of 43 to 87.5 kg; mean physical activity level was 357 kcal/d with a range of 3 to 1543 kcal/d. Serum 25‐hydroxyvitamin D concentrations did not differ among the groups for the 180 participants in whom it was available (60 participants the 90‐Hz group, 61 in the 30‐Hz group, and 59 in the control group), with an overall mean concentration of 93 nmol/L (SD 29). There was no significant difference in the changes over the 12 months in physical activity levels or total daily vitamin D intake among the intervention groups (data not shown).

**Table 1:** Baseline Characteristics of Participants

Characteristic	Participants randomized to WBV (30 or 90 Hz) (*n* = 135)	Participants randomized to no WBV (control) (*n* = 67)
Mean age, years (SD)	60.0 (6.5)	60.8 (5.5)
Ethnicity, *n* (%)		
European	103 (76)	54 (81)
Southeast Asian	22 (16)	10 (15)
Other	10 (7)	3 (4)
Smoking, *n* (%)		
Never smoked	84 (62)	43 (64)
Former or current smoker	51 (38)	24 (36)
Mean body mass, kg (SD)	63.2 (10.6)	62.4 (9.5)
Mean height, m (SD)	1.60 (0.06)	1.60 (0.06)
Mean BMI, kg/m^2^ (SD)	24.7 (3.8)	24.2 (3.4)
Mean physical activity metabolic index, kcal/d (SD)	345 (230)	383 (277)
Falls experienced in past 12 months, *n* (%)		
None	106 (79)	48 (72)
≥1	29 (21)	19 (28)
Mean vitamin D intake, IU/day (SD)	821 (582)	808 (584)
Mean distal tibia HR‐pQCT bone total BMD, mg/cm^3^ (SD)	266.1 (44.5)	263.7 (43.4)

WBV = Whole‐body vibration.

Complete adherence data was previously published.^23^ Adherence to WBV was similar in the 30‐Hz and 90‐Hz WBV groups with a median adherence of 65% to 79% for the three measures of cumulative duration, number of days, and full session counts.^23^

### Myotendinous outcomes

**Figure 3:**
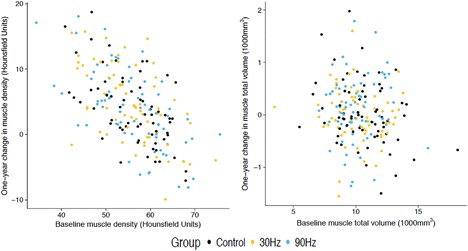
Baseline and 12‐month increase in myotendinous density and volume.

There was no significant difference in mean change in M_T_D and M_T_V from baseline to 12 months between the three intervention groups (*p* = 0.70 for M_T_D, *p* = 0.50 for M_T_V; Table 2). Single imputation was used to replace missing outcomes on 10 subjects (4.9%) at follow‐up. As seen in Fig. 3, the 12‐month change in myotendinous outcomes did not differ among the three randomized groups. Specific comparisons of groups (30‐Hz WBV versus control, 90‐Hz WBV versus control, and 90‐Hz WBV versus 30‐Hz WBV) also revealed small non‐statistically significant differences in the effect of treatment on M_T_D and M_T_V (Table 2) with CIs that excluded differences between groups of more than one‐fifth of a baseline SD for M_T_V and one‐third of a SD for M_T_D.

**Table 2:** Mean Changes in HR‐pQCT Myotendinous Density, Volume, and Timed‐Up and Go (TUG) From Baseline to 12 Months and Between‐Group Differences From the Baseline‐Adjusted ANCOVA

				ANCOVA‐adjusted mean differences with 95% CI		
Measurement	30‐Hz WBV group (*n* = 68)	90‐Hz WBV group (*n* = 67)	Control group (*n* = 67)	30‐Hz WBV group versus Control group	90‐Hz WBV group versus Control group	90‐Hz WBV group versus 30‐Hz WBV group
Myotendinous density (M_T_D)						
Baseline myotendinous density, HU (SD)	55.7 (7.3)	55.9 (8.1)	56.2 (6.8)			
12‐month myotendinous density, HU (SD)	60.3 (6.1)	59.8 (6.4)	60.0 (5.7)			
Mean change in myotendinous density, HU (SD)	4.6 (5.7)	3.9 (6.1)	3.9 (5.4)	0.5 (−1.4 to 2.4)	−0.1 (−2.0 to 1.8)	−0.6 (−2.5 to 1.2)
Myotendinous volume (M_T_V)						
Baseline myotendinous total volume, mm^3^ (SD)	10149 (1879)	10125 (1611)	10632 (2145)			
12‐month myotendinous total volume, mm^3^ (SD)	10142 (1908)	10237 (1756)	10667 (2048)			
Mean change in myotendinous total volume, mm^3^ (SD)	−7 (503)	112 (615)	35 (615)	−60 (−296 to 175)	57 (−180 to 293)	118 (−116 to 352)
TUG						
Mean baseline TUG, s (SD)	6.1 (1.0)	6.0 (1.0)	6.1 (1.0)			
Mean 12‐month TUG, s (SD)	5.7 (0.9)	5.6 (0.9)	5.8 (0.9)			
Mean absolute change in TUG, s (SD)	−0.4 (0.7)	−0.3 (0.9)	−0.3 (0.9)	−0.0 (−0.4 to 0.3)	−0.1(−0.4 to 0.3)	−0.0 (−0.4 to 0.3)

Results of per‐protocol analyses (with only subsets of participants in the WBV groups meeting various thresholds of adherence) were similar to those from ITT analysis and showed no significant effect of WBV therapy (data not shown). Results of analysis of participants with differing adherence (participants with adherence threshold >50%, >60%, >70%, and >80% adherence) are similar, with no significant treatment effects on M_T_D and M_T_V, even when restricted to those with a high adherence of >80% (data not shown). There were no significant interactions between baseline covariates and the effect of WBV on M_T_D and M_T_V, whether analyzed as continuous variables or as dichotomous variables (data not shown).

### Other outcomes

TUG measurements were missing on 34 participants at baseline, follow‐up, or both because participants declined to perform the test or were lost to follow‐up. There was no significant change in TUG between intervention groups for those with complete data (*p* = 0.93) or when multiple imputation was used to account for the 15% of subjects with missing baseline or follow‐up TUG (*p* = 0.95). Pairwise differences between treatment groups were small (Table 2). There was also no significant change in body mass or BMI among the treatment groups over the study period (data not shown).

There was no significant difference among the groups in the number of participants who had a clinical fracture during the study, and no participants had fragility fractures (4 participants with fractures in the 90‐Hz group, 1 participant in the 30‐Hz group, and 1 participant in the control group, *p* = 0.33). Twenty‐one participants (31.3%) in the 90‐Hz WBV group, 12 (17.7%) in the 30‐Hz WBV group, and 24 (35.5%) in the control group had one or more falls (*p* = 0.045 comparing all three groups). Although there were fewer falls in the 30‐Hz WBV group than in the other two groups, none of the pairwise differences were statistically significant when adjusted for multiple comparisons (smallest *p* = 0.085).

As assessed by a blinded investigator, there were no serious adverse events that were thought to be related to WBV. WBV therapy was well‐tolerated in the study and adverse events did not differ statistically among study groups. Adverse events and symptoms attributed to WBV by the participants have been reported in full.^23^

## Discussion

This is the first study to assess the effect of WBV at the clinically relevant distal lower extremity and ankle site using HR‐pQCT. In this 12‐month trial of 202 postmenopausal women randomized to control or daily low‐magnitude WBV at 30 or 90 Hz, we did not observe the hypothesized beneficial effects of WBV on M_T_D and M_T_V at the distal leg as measured by HR‐pQCT. Furthermore, the 95% CIs comparing mean 12‐month M_T_D and M_T_V between the three groups were narrow enough to exclude differences on the order of one‐quarter of a baseline SD, a value generally accepted as a small effect size.

Our findings are in contrast to previous studies that have examined muscle outcomes with different imaging modalities (CT or whole‐body DXA) and have found significant changes with WBV.^15^, ^16^, ^17^ In theory this discrepancy could be explained by the method and location of assessment of M_T_D and M_T_V. In the current study, we measured M_T_D and M_T_V at the distal leg using HR‐pQCT. The lower leg was chosen as it is the site of the muscle and tendons involved in ankle plantar flexion, which is important to balance and stability and in the prevention of falls.^34^ The lower leg is also the site most proximal to the WBV plate while standing on it; thus, this site would theoretically have greater vibration propagation effect than the proximal thigh region.^8^ Further, the degree of proximal vibration propagation from the plate up the lower extremity is also in part related to the participants’ posture–flexed hips and knees versus extended hips and knees. Therefore, it follows that we would expect changes in the M_T_D and M_T_V at the distal tibia region that would correlate with changes at muscle sites further from the transmission of the vibrations, such as the upper thigh as previously studied,^15^, ^16^ if such changes are produced. The studied area contains myotendinous junctions (important biomechanical structures); thus, changes in M_T_V at this site would be physiologically significant.

Tendon mechanical properties and calf muscle size decrease in older adults, along with balance indices; thus the observed decreases in muscle strength with aging are not solely based on changes in muscle size, but also tendons.^35^, ^36^ Muscle and myotendinous tissue density and volume were measured as they have been shown to relate to functional measurements such as specific force, falls, and functional impairment and disability such as walking, climbing stairs, and performing activities of daily life.^3^, ^10^, ^22^ Different CT modalities have been used to assess muscle, including pQCT.^37^ Though assessing muscle and myotendinous outcomes with HR‐pQCT is a novel application, our research group has previously demonstrated that myotendinous volume at the distal site as measured by HR‐pQCT moderately correlates with commonly assessed pQCT measures of lower limb muscle density and area in postmenopausal women.^27^

Another factor that could explain the observed discrepancy with previous literature is the type of WBV therapy we used. In our study, participants stood without movement on the WBV platform for 20 min daily. In previous studies showing an effect of WBV on muscle outcomes, exercises such as squatting were done during the WBV.^15^, ^16^, ^17^ It is possible that static and dynamic exercises, posture including the degree of hip and knee flexion, protein supplementation, or the combination of exercises while on the WBV platform, are critical to elicit an effect on muscle and myotendinous outcomes.

A meta‐analysis of WBV in older adults has found that the effect on falls reduction remains uncertain.^12^ A recent randomized controlled trial comparing 6 weeks of WBV therapy plus exercise to exercise alone, reported no effect on the number of falls.^38^ Our finding of no significant effect on TUG scores is in contrast with two previous meta‐analyses,^12^, ^14^ but in agreement with another meta‐analysis.^13^ In our study, TUG was at most 0.4 s less in 90‐Hz WBV compared with control at follow‐up, with TUG decreasing in all groups at 12 months compared with baseline. At baseline, all three groups were performing the TUG in the normal range (mean 6.1 s in 30‐Hz group, 6.0 s in 90‐Hz group, and 6.1 s in control group). As the TUG values were normal to begin with, it is possible no effect was observed as there was limited room for improvement (ceiling effect).

Although this was a rigorously designed and conducted randomized controlled trial, there are limitations to our study. The study was not able to blind receipt of WBV because of a lack of funding and an impracticality for sham platforms. Our participants were relatively healthy and motivated, which may have resulted in limited room for improvement in muscle outcomes, although it is more likely that the posture we chose for participants to assume during WBV was not optimal for assuring therapeutic efficacy for myotendinous outcomes. The chosen myotendinous outcomes were secondary exploratory outcomes of the Vibration Study; as such, we were not able to look at muscle at other sites. The clinical significance of myotendinous properties at the distal tibia on clinical outcomes, such as mobility, balance, and falls, has not been proven and is currently being investigated by our group. One strength of our trial is the duration of the intervention; most published studies evaluating WBV as an alternate to therapeutic exercise are typically a maximum of 3 months in duration. Adherence was not uniformly high, which may have limited our ability to detect an effect of the interventions, but this also reflects “real‐world” practice with WBV or exercise. Several participants did not have baseline and follow‐up TUG testing; however, given the low average TUG time for our sample, it is unclear if the missing data would have altered our findings.

In conclusion, 12 months of daily 30‐ or 90‐Hz WBV therapy had no effect on myotendinous density or volume at the distal leg as measured by HR‐pQCT. Not only were the differences not statistically significant, but their CIs were narrow enough to rule out anything but a clinically small effect. Further high‐quality research is needed to prospectively determine the type of therapies that may affect myotendinous parameters at the lower extremities, intermediate outcomes such as strength and balance, and clinical outcomes such as falls.

## Disclosures

LMG has received consulting fees or honorarium from Icon and Eli Lilly.
